# MicroRNA-146b: A Novel Biomarker and Therapeutic Target for Human Papillary Thyroid Cancer

**DOI:** 10.3390/ijms18030636

**Published:** 2017-03-15

**Authors:** Chen-Kai Chou, Rue-Tsuan Liu, Hong-Yo Kang

**Affiliations:** 1 Division of Endocrinology and Metabolism, Department of Internal Medicine, Kaohsiung Chang Gung Memorial Hospital, Chang Gung University College of Medicine, Kaohsiung City 833, Taiwan; chou@adm.cgmh.org.tw (C.-K.C.); ruetsuan@ms2.hinet.net (R.-T.L.); 2 Graduate Institute of Clinical Medical Sciences, Chang Gung University, Kaohsiung City 833, Taiwan; 3 Hormone Research Center and Department of Obstetrics and Gynecology, Kaohsiung Chang Gung Memorial Hospital, Chang Gung University College of Medicine, Kaohsiung City 833, Taiwan

**Keywords:** papillary thyroid carcinoma, microRNA-146b, target gene, tumorigenesis

## Abstract

Papillary thyroid cancer (PTC) is the most common tumor subtype of thyroid cancer. However, not all PTCs are responsive to current surgical and radioiodine treatment. The well-established clinical prognostic factors include tumor size, lymph node/distal metastasis, and extrathyroidal invasion. The *RET*/PTC-*RAS*-*BRAF* linear molecular signaling cascade is known to mediate PTC pathogenesis. However, whether presence of *BRAF* mutation, the most common genetic alteration in PTC, can affect PTC behavior and prognosis is controversial. MicroRNAs (miRNAs) have been labeled as promising molecular prognostic markers in several tumor types. Our recent studies demonstrated that microRNA-146b (miR-146b) deregulation is associated with PTC aggressiveness and prognosis. Here we summarize the current knowledge related to the functional roles, regulated target genes, and clinical applications of miR-146b in PTC and discuss how these studies provide insights into the key role of miR-146b as an oncogenic regulator promoting cellular transformation as well as a prognosis marker for tumor recurrence in PTC. In conjunction with the current perspectives on miRNAs in a wide variety of human cancers, this review will hopefully translate these updated findings on miR-146b into more comprehensive diagnostic or prognostic information regarding treatment in PTC patients before surgical intervention and follow up strategies.

## 1. Introduction

The prevalence of thyroid cancer has increased over time from the worldwide reports; the majority of thyroid cancers are papillary thyroid carcinoma (PTC), which is clinical-pathogenically heterogeneous in tumor behavior or prognosis [1,2]. Although most of PTCs could be managed successfully with a combination of radioactive iodide and levothyroxine suppression therapy after complete surgical intervention, a certain proportion of PTCs remain irresponsive to treatment and result in comorbidity and mortality. Therefore, it is crucial to further elucidate the pathogenesis of PTC regarding tumor behavior at the molecular level.

In PTCs, rearrangements in *RET*/PTC and *NTRK* and point mutations in *RAS* and *BRAF* constitutively activate the mitogen-activated protein kinase (MAPK) pathway, which are cumulatively found in over 70% PTCs [3,4,5]. Numerous clinicopathological and molecular studies provide compelling genetic evidence that constitutively active MAPK signaling is a key component of thyrocyte transformation in PTC. The most common genetic alteration among these mutations is the *BRAF*^V600E^ point mutation, which is demonstrated to be significantly associated with extrathyroidal invasion, advanced disease stages, lymph node metastasis, and tumor recurrence in PTC [6]. However, not all studies support the assessment that the *BRAF*^V600E^ point mutation is an independent predictor of the disease extent or outcome. Some studies find it not to be predictive at all [7], some find it significant only in univariate but not in multivariate analysis [8], whereas others find it to be useful in addition to other specific clinical and histological features [9]. However, Sancisi et al. [10] studied *BRAF*^V600E^ mutation in metastatic PTC and found that the *BRAF*^V600E^ mutation was present in 29.8% of the distantly metastatic PTC, whereas it was detected in about 44.0% of the control tumors, suggesting that the *BRAF* mutation was of no significance for active disease progression. Moreover, Guerra et al. [11] demonstrate that clonal *BRAF*^V600E^ is a rare occurrence in PTC suggesting that the *BRAF*^V600E^ mutation is a late subclonal event in PTC. This evidence supports the concept that the presence of *BRAF* mutation may not be the fundamental event in tumorigenesis or an independent predictor of outcome. Therefore, identification of a new marker associated with *BRAF*-positive tumors and poor prognosis is required and may serve as a new therapeutic target and follow-up marker in PTC.

MicroRNAs (miRNAs) are small noncoding RNA molecules that function as negative regulators of gene expression by binding to the 3′-untranslated region of candidate mRNAs, and blocking the translation or degradation of target mRNAs that regulate various pathophysiological courses [12]. Regulation of classical oncogenes and tumor suppressor genes by miRNAs was effortlessly identified as a hallmark of cancer research, transforming this class of small RNAs into potential targets for cancer therapy, diagnosis, and prognosis. Recent studies on miRNA deregulation have demonstrated increased aberrant miRNA expression (particularly, miR-222, miR-221, and miR-146b) in PTCs compared to that in normal thyroid tissues [13,14,15]. These data indicated the distinct miRNA profile associated with PTC carcinogenesis. Furthermore, the deregulation or aberrant expression of several miRNAs in PTC has been implicated in disease development, progression, and prognosis [13,14,16,17,18]. A microRNA array study [19] demonstrated that deregulation of microRNA-146b (miR-146b) was significantly associated with aggressive tumor behavior in *BRAF*-positive clinical PTC specimens. In the context of *BRAF* mutation and miR-146b, we noticed that patients harboring *BRAF* mutations had a higher expression level of miR-146b than that in *BRAF* wild-type patients [15]. Furthermore, Geraldo et al. [20] demonstrated that transformation of *BRAF* mutation resulted in significant elevation of miR-146b levels. The discovery of *BRAF*-miRNA regulation opens a new perspective in the understanding of PTC biology.

## 2. Structure, Expression, and Regulation of MicroRNA-146b

**Figure 1:**
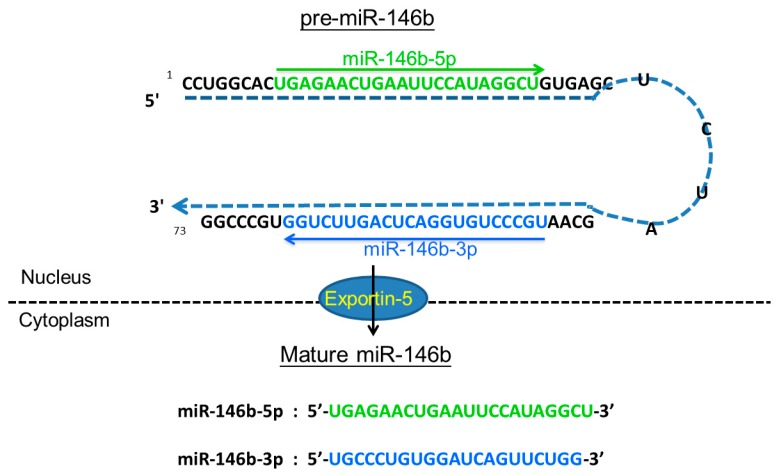
The hairpin structure of pre-miR-146b and the sequence of mature miR-146b. The *MIR146B* gene is located in an intergenic region of chromosome 10q24.32 and transcribed into a precursor (pre-miR-146b) with 73 nucleotides in the nucleus, that is exported to the cytoplasm by Exportin-5 for additional processing to yield two mature microRNAs with 22 nucleotides, miR-146b-5p and miR-146b-3p. The sequence of mature miR-146b-5p and miR-146b-3p is colored in green and blue. The arrow shows the orientations from 5′ to 3′.

MiR-146a and miR-146b are known as post-transcriptional gene silencers, which play an important role in regulating inflammatory responses [21]. They also share identical sequences except for a difference of two nucleotides in the 3′-end and hence could translationally down-regulate similar candidate genes. Mature forms of miR-146a and miR-146b are encoded by two separate genes—*MIR146A* and *MIR146B*—localized to human chromosomes 5 and 10, respectively. The stem-loop structure of pre-miR-146b and the sequences of mature miR-146b are shown in Figure 1. It is known that miRNAs can be regulated by multiple genes or factors, sequentially or simultaneously, and previous studies have identified the up-stream regulators of miR-146b [21,22]. While miR-146b has also been demonstrated as one of the downstream targets of nuclear factor-κB (NF-κB) signaling and is up-regulated by toll-like receptor ligand treatment as well as in response to tumor necrosis factor α or interleukin-1β stimulation [21]. Furthermore, PDGF (platelet-derived growth factor) regulates the transcription of miR-146b and provides evidence for a miR-dependent feedback mechanism balancing growth factor receptor signaling in cancer cells [22].

Differential miR-146b expression between normal thyroid tissue and PTC has been shown previously [13,15,17,23,24,25]. On the basis of histological features, emerging evidence indicates that dysregulated miR-146b is implicated in the different variants of PTC. PTC is a very heterogeneous group of tumors that can be further categorized into several subtypes: such as classical variant of PTC (cPTC), follicular variant of PTC (fvPTC), and tall cell variant (tcPTC). A recent study analyzed the datasets of 466 thyroid cancer samples sequenced in The Cancer Genome Atlas (TCGA) and showed that the expression level of miR-146b is higher in cPTC than fvPTC, but no significant difference was noted between cPTC and tcPTC [26]. The similar expression level of miR-146b in infiltrative fvPTC and noninvasive follicular thyroid tumor with papillary-like nuclear features (NIFTP) was also reported recently [27].

A genomic, epigenomic, and proteomic profiling of a large cohort of PTC patients reveals that several microRNAs including miR-146b are classified as oncomirs, which are associated with metastasis [28]. Several studies have suggested that elevated miR-146b expression may play a role in advanced malignant tumor characteristics [15,19,24,29,30,31], including extra-thyroidal invasion and advanced stages of PTC. Furthermore, several recent studies, including our own, have provided evidence that miR-146b overexpression even plays a critical role in PTC progression and patient prognosis [32,33]. Overexpression of miR-146 has also been observed in follicular thyroid carcinoma (FTC) [34] and poorly differentiated thyroid carcinoma [35], suggesting that this miRNA is full of potential to be further investigated its pathogenesis roles in the malignant thyroid neoplasms.

## 3. Cellular Functions of miRNA-146b in Papillary Thyroid Carcinoma (PTC)

Despite significant progress in the knowledge about miR-146b deregulation in thyrocyte transformation, there is much more to elucidate about the signal transduction of miR-146b regulatory mechanisms and their functional impact in PTC cells. A number of studies related to the roles of miR-146b-regulated genes in enhancing PTC behavior are listed in Table 1. Geraldo et al. [20] reported that overexpression of miR-146b suppressed *SMAD4* (SMAD family member 4) expression, thereby leading to up-regulated resistance against TGF-β-mediated cell cycle arrest in PTC cell line. Deng et al. [36] demonstrated the involvement of miR-146b-*ZNRF3* signal transductions in epithelial-mesenchymal transition (EMT) through modulation of Wnt/β-catenin signaling. In order to delineate the role of miR-146b in EMT by augmenting PTC cancer cell migration/invasion, we identified a regulatory mechanism linking miR-146b and its target gene *IRAK1* in PTC cell lines [37]. The function of the miR-146b-*IRAK1* axis may be potentially associated with EMT by regulation of E-cadherin in PTC cancer cell lines. We also confirmed that miR-146b promotes aggressive tumor characteristics in PTC by suppressing *IRAK1* expression and that restoration of *IRAK1* expression reversed this outcome. In agreement with our findings, Lima et al. [38] reported that miR-146b positively regulates migration and invasion activity both in normal or cancer thyroid lines through the action of stimulating actin cytoskeleton functions. In addition, miR-146b and its related target genes play an important role in other thyroid neoplasms. The expression level of miR-146b has been proved to be negatively regulated by *HDAC3* and inhibition of miR-146b expression increased the radioactive iodide sensitivity by regulating the sodium/iodide symporter (*NIS*) expression in poorly differentiated thyroid cancer cells [35]. Riesco-Eizaguirre et al. [39] stated that miR-146b binds to the 3′-untranslated region of *PAX8* and *NIS*, thus repressing iodide protein translation and leading to insensitivity to radioactive iodide treatment. Wang et al. [40] reported that p21 is regulated by miR-146b and leads to tumor proliferation and migration in anaplastic thyroid cancer (ATC) cells. However, there are conflicting reports for miR-146b in other solid neoplasms. Bhaumik et al. [41] found that miR-146b expression suppresses NF-κB activity and reduces metastatic potential in human breast cancer cell lines. Moreover, the expression of epidermal growth factor receptor (EGFR) in human glioblastoma cell lines was suppressed by miR-146b, which reduced their migration and invasion in vitro [42]. While the activity and biological function of miR-146b behaving either as an oncogene or tumor suppressor depending on different types of cancer is not well-understood, it is conceivable that the miR-146b may interact with different cancer-specific cellular and molecular context to regulate its specific target genes and to determine the functions of miRNA–target interaction regulatory networks in specific tumor microenvironment.

## 4. Clinical Applications of MicroRNA-146b in PTC

It is believed that miRNAs are more stable than mRNA, thus the clinical application of miRNA detection can be easily used in clinical specimens like plasma or serum. An emerging number of studies [17,43,44,45,46] have reported that several distinct miRNA panels, which include miR-146b, could distinguish malignant tissues from benign lesions in thyroid fine-needle aspiration (FNA) specimens via quantitative polymerase chain reaction. Furthermore, circulating miR-146b expression was demonstrated as a convenient and useful serological marker to discriminate between PTC and benign lesions or healthy individuals [47,48]. Detection of miR-146b expression by in situ hybridization analysis has also shown unique diagnostic value in distinguishing PTC from FTC and ATC [49]. Furthermore, The Cancer Genome Atlas (TCGA) has suggested that the potential miR-146b-*IRAK1* regulation characterizes classic PTC subtype [26].

Although clinical applications concerning miR-146b levels in pre-operation thyroid tissue remain unclear, our study demonstrated the prognostic prediction value of miR-146b expression by long term follow up [32]. Furthermore, in patients undergoing prophylactic central neck lymph node dissections, miR-146b is identified as potential markers of central neck lymph node metastasis [50]. Thus, the status of miR-146b expression appears to have supplementary diagnostic value and provides more comprehensive information regarding patient’s condition before surgical intervention. The clinical applications of miR-146b in PTC are shown as Table 2.

## 5. Conclusions

The accumulating reports raise an appealing concept that sequence-specific inhibition of miRs in stem/progenitor cell populations can deliver a potential therapeutic strategy for modulation of stem/progenitor cells whose miRs are deregulated in cancer. In the study by Hardin et al., PTC cells acquired increased cancer stem cell-like features and the expression of miR-146b and *PRRX1*, an EMT marker, was markedly up-regulated [51]. Knockdown of miR-146b had an inhibitory role during TGF-β1-induced EMT, suggesting therapeutic potential for such modulation. It is likely that modulation of miR-146b may sensitize stem/progenitor cells in aggressive PTC that remain resistant to the treatment of radioactive iodide and levothyroxine suppression therapy after complete surgical intervention. It will be of great interest to investigate whether targeting miR-146b in PTC is one of the key approaches that enhance susceptibility of cancer stem/progenitor cells to radio-therapeutic treatments. Clearly, understanding the molecular basis involved in the development and aggravation of PTC will be helpful for identifying novel diagnostic, prognostic, and therapeutic targets. Although the exact mechanisms and clinical applications of miR-146b are yet to be fully elucidated, miR-146b expression in PTC not only provides a unique supplemental tool for diagnosis and predicting prognosis, it may also serve as a novel biomarker and therapeutic target for PTC in the near future.

## Figures and Tables

**Table 1:** Summary of miR-146b regulatory molecules and their effects in thyroid cancer cell lines.

Regulatory Molecule/Pathway	Action	Function	Direct/Indirect	Cancer Cell Line	Cancer Subtype	Reference
Downregulated *SMAD4*	Inhibit TGF-β anti-signal	Increase proliferation activity Inhibit cell cycle arrest	Direct	TPC-1 and BCPAP	PTC	[20]
Downregulated *NIS*	*HDAC3* suppresses miR-146b	Decrease sensitivity to radioactive iodide	Direct	FTC-133	Poorly differential thyroid carcinoma	[35]
Downregulated *ZNRF3*	Mediated by Wnt/β-catenin signaling	Migration, invasion and EMT	Direct	TPC-1 and K1	PTC	[36]
Downregulated *IRAK1*	Associated with EMT process	Increase migration, proliferation	Direct	BCPAP and TPC-1	PTC	[37]
Downregulated *PAX8* and *NIS*	Decrease iodide protein translation	Decrease sensitivity to radioactive iodide	Direct	PCCl3	PTC	[39]
Upregulated p21	Cell cycle progression	Increase migration and proliferation	Indirect	FRO	ATC	[40]

Papillary thyroid cancer; ATC, anaplastic thyroid cancer; FTC, follicular thyroid carcinoma; EMT, epithelial-mesenchymal transition.

**Table 2:** Clinical applications of highly expressed miR-146b in PTC.

Mode of Implication	Specimens	Experiments	Reference
Predicts the poor prognosis	Thyroid cancer tissue	Quantitative polymerase chain reaction	[32]
Differentiates malignancy from benign lesions	Thyroid fine needle aspiration	Quantitative polymerase chain reaction	[43,44,45]
Distinguishes between benign and malignant	Plasma	Quantitative polymerase chain reaction	[47]
Acts as biomarkers for the PTC recurrence	Thyroid cancer tissue and plasma	Quantitative polymerase chain reaction	[48]
Distinguishes PTC from FTC and ATC	Thyroid cancer tissue	In Situ Hybridization Analysis	[49]
Characterizes classic PTC subtypes	Thyroid cancer tissue	Quantitative polymerase chain reaction	[26]
Predicts central neck lymph node metastasis preoperatively	Thyroid cancer tissue	Quantitative polymerase chain reaction	[50]

## Abbreviations

ATC	Anaplastic thyroid cancer
cPTC	Classical variant of PTC
fvPTC	Follicular variant of PTC
EGFR	Epidermal growth factor receptor
FTC	Follicular thyroid carcinoma
FNA	Fine-needle aspiration
EMT	Epithelial-mesenchymal transition
miRNAs	MicroRNAs
MAPK	Mitogen-activated protein kinase
NIFTP	Noninvasive follicular thyroid tumor with papillary-like nuclear features
NF-κB	Nuclear factor-κB
PTC	Papillary thyroid cancer
PDGF	Platelet-derived growth factor
*NIS*	Sodium/iodide symporter
